# Intersection of anxiety and negative coping among Asian American medical students

**DOI:** 10.3389/fpsyg.2022.929227

**Published:** 2022-09-02

**Authors:** Michelle B. Moore, David Yang, Amanda M. Raines, Rahn Kennedy Bailey, Waania Beg

**Affiliations:** ^1^LSU Health Sciences Center New Orleans, Louisiana State University, New Orleans, LA, United States; ^2^Department of Emergency Medicine, Yale University, New Haven, CT, United States; ^3^Southeast Louisiana Veterans Health Care System, Veterans Health Administration, United States Department of Veterans Affairs, New Orleans, LA, United States

**Keywords:** anxiety, negative coping, Asian American, medical students, intersection of anxiety

## Abstract

**Purpose:**

Asian Americans comprise 21% of matriculating medical students in the United States but little is known about their mental health. With the growing focus on addressing the mental health of medical students, this systematic, nationwide survey assesses the relationship between anxiety and depression symptoms and coping skills among Asian American medical students.

**Materials and methods:**

A survey tool comprised of Patient Health Questionnaire-9, General Anxiety Disorder-7, and questions related to coping were emailed to members of the Asian Pacific American Medical Students Association enrolled in a United States medical school during the 2016–2017 academic year. We evaluated associations between anxiety and coping as well as depression and coping.

**Results:**

A total of 511 Asian American medical students completed the survey. Anxiety symptoms were positively correlated with an increase in negative coping skills. Depressive symptoms were not correlated with an increase in negative coping skills.

**Conclusion:**

Professionals and medical schools that aim to improve the mental health of medical students should be aware of the needs of specific populations. Asian American students who experience anxiety were more likely to utilize avoidant or negative coping strategies. In addition, Asian American students who experience depressive symptoms were not more likely to utilize these negative coping strategies. Further research must be done to evaluate the factors that influence the use of negative coping strategies to better address anxiety within the Asian American medical student population.

## Introduction

Individuals who identify as Asian American comprise 21% of matriculating medical students in the United States, but there is limited research focusing on the mental health of these students throughout their medical school experience (Dyrbye et al., 2007; Association of American Medical Colleges, 2021). While there are several studies that have looked at stress, depression, and burnout among minority medical students in general (Pyskoty et al., 1990; Camp et al., 1994; Henning et al., 1998; Tjia et al., 2005; Dyrbye et al., 2006a,^2007^; Yang et al., 2021), there are fewer studies also examining anxiety among this population and, in particular, a paucity of data on medical students who identify as Asian American (Quek et al., 2019). Existing literature examining the prevalence of anxiety and depression among medical students found that medical students had higher rates of anxiety and depression relative to the general population (Dyrbye et al., 2006b). Anxiety and depressive disorders can negatively impact medical students’ health, professionalism, academic performance, and quality of patient care (Burr and Beck Dallaghan, 2019; Quek et al., 2019). Given that Asian American medical students comprise almost a quarter of the students attending medical school, understanding what cultural considerations need to be made to address this population of students is important to consider prior to their completion of training when they are entering the workforce as physicians. Cultural considerations, which take into consideration how stigma related to seeking mental health services and acknowledging symptoms that could lead to emotional stress and burnout, could positively impact interventions and conversations with Asian American medical students regarding their mental health needs while in medical school. Therefore, it is imperative for medical school administrators to understand the consequences of both anxiety and depression on Asian American medical students.

The general population of medical students use a variety of coping skills to manage anxiety and depression (Stern et al., 1993). Coping skills refer to the behavioral and cognitive efforts individuals employ in response to stressors in an effort to minimize the negative implications associated with a stressor (Merrill and Thomas, 2013). A distinction is often made between positive and negative coping skills (de La Rosa-Rojas et al., 2015). Positive or adaptive coping are defined as an active approach that aims to mitigate or change the stressor itself through a means that is beneficial to the overall wellbeing of that individual (Folkman et al., 1986). For instance, contacting a therapist or counselor, exercising, and seeking support from spiritual advisors are typically identified as positive coping skills (Litman, 2006). In contrast, negative or avoidant coping is a passive approach centered on escaping from stressful situations and subsequently often harmful to the individuals wellbeing (Merrill and Thomas, 2013). Engaging in self-harm, over or under-eating, and consuming alcohol are commonly identified as negative coping skills (Litman, 2006). Positive coping skills have been linked to better health outcomes than negative coping, such as lower levels of stress symptoms, decreased depression, and decreased suicidal behavior (Penley et al., 2002; Li and Zhang, 2012; Merrill and Thomas, 2013; Shatkin et al., 2016; Thompson et al., 2016; Nie et al., 2020). Conversely, negative coping skills have been associated with greater alcohol consumption, lower quality of life, burnout, fatigue, and increased risk of mental health problems (Merrill and Thomas, 2013; Ding et al., 2015; Paek et al., 2016; Hou et al., 2020; Smida et al., 2021).

Previous studies in medical students have shown an association between coping skills and depression and anxiety symptoms (Mosley et al., 1994; Steiner-Hofbauer and Holzinger, 2020). Shao et al. (2020) reported that anxiety was negatively correlated to positive coping and positively correlated to negative coping in Chinese medical students. A German study found that students with high scores on anxiety and depression clinical scales also showed high levels of dysfunctional coping (Prinz et al., 2012). Thompson et al. (2016) also reported that greater use of positive or approach-oriented coping strategies, which aim to find cognitive strategies to find a solution to a problem, was inversely correlated with depression. To our knowledge, data on anxiety, depression, and coping strategies of Asian American medical students in the United States are lacking. The intersection of these areas is important for medical schools to understand in order to build culturally sensitive interventions that can lead to a healthy and sustainable workforce after medical school. As previous studies have shown, individuals who identify as minority students often experience unique challenges in how they utilize coping skills to manage mental health symptoms. In addition, Asian American students are often considered the “model minority,” which possesses additional stressors on individuals to perform and achieve high standards. This model minority myth, which creates an assumption that one minority group is perceived as higher achieving over other individuals due to their ethnicity, creates misperceptions regarding one’s true mental health needs by creating false assumptions regarding the Asian American population (Cheng et al., 2017; Yip et al., 2021). Thus, the aim of this study was to explore the relationship between anxiety and depression symptoms and coping skills among Asian American medical students attending medical schools in the United States.

## Materials and methods

### Participants and procedures

The sample included 511 Asian American (AA) medical students enrolled at a medical school in the United States during the 2016–2017 academic year. Students were recruited through the Asian Pacific American Medical Student Association, a national organization that supports AA students and represents 90 medical schools and colleges in the United States. Specifically, students were emailed information regarding the study and invited to complete a brief, online battery of self-report questionnaires. The survey was comprised of questions regarding coping behaviors, the Generalized Anxiety Disorder-7 self-report questionnaire, the Patient Health Questionanire-9 self-report measure, questions related to stigma, mental health history and demographic questions. All questions were included to further understand mental health symptoms, behaviors and attitudes for the respective sample population. All responses were completely anonymous and collected using the professional and encrypted version of Survey Monkey Pro. The study was approved by the institutions’ Institutional Review Board.

Students’ who completed the survey identified as primarily male (62%) and between the ages of 21–25 (68%). In terms of relationship status, most students reported being single (49%) followed by married or in a committed relationship (51%). Most students were enrolled in their first year of medical school (40%) followed by second (31%), third (19%), and fourth year (10%).

### Measures

#### Coping

Participants were provided with a list of behaviors and asked to select all methods that are used to cope when feeling anxious or depressed. Negative coping behaviors, included the following strategies which were engaging in self-harm, overeating, undereating, smoking cigarettes, using recreational drugs, and consuming alcohol. The negative coping strategies were coded as 1. The score for each individual was summed to create a total score (ranging from 0 to 6) with higher scores indicating poorer coping.

#### Generalized anxiety

The Generalized Anxiety Disorder-7 (GAD-7) is a 7-item self-report questionnaire designed to assess generalized anxiety symptoms (Spitzer et al., 2006). Participants were asked to read a list of symptoms and indicate how often they have been bothered by each within the past 2 weeks. Items are rated using a four-point Likert-type scale ranging from 0 (*Not at all*) to 3 (*Nearly every day*). Total scores can range from 0 to 21 with higher scores indicating increased symptom severity. The GAD-7 has strong psychometric properties and demonstrated good internal consistently in the current study (Cronbach α = 0.88).

#### Depression

The Patient Health Questionnaire-9 (PHQ-9) is a 9-item self-report questionnaire designed to assess depression symptoms. Participants were asked to read a list of symptoms and indicate how often they have been bothered by each within the past 2 weeks (Kroenke et al., 2001). Items are rated using a four-point Likert-type scale ranging from 0 (*Not at all*) to 3 (*Nearly every day*). Total scores can range from 0 to 27 with higher scores indicating increased symptom severity. The PHQ-9 has strong psychometric properties and demonstrated good internal consistently in the current study (Cronbach α = 0.87).

#### Drop out

Participants were also asked if they had considered dropping out of medical school over the past month using a dichotomous response choices of *Yes* or *No*.

### Data analytic plan

All analyses were conducted using IBM SPSS Statistics version 26.0. First data screening was performed. This included calculating descriptive statistics to inspect for data entry errors, missing data, outliers, and normality. Normality, linearity, and homoscedasticity were assessed according to the guidelines sets forth by Tabachnick and Fidell by inspecting the normal probability plot of the regression standardized residual and the scatterplot as well as tolerance and variance inflation factor values (Tabachnick and Fidell, 2006). Second, descriptive statistics including means, standard deviations, and distributions were examined. Variables were considered abnormally distributed if the skewness and/or kurtosis statistic divided by the respective standard error resulted in a value ±2. In such cases a Log10 transformation was utilized. Third, zero-order correlations among all variables were examined. Fourth, two hierarchical linear regression analyses were conducted to examine the relationship between anxiety, depression, and poor coping. Gender was entered in the first step of each model followed by either anxiety or depression symptom severity.

## Results

### Preliminary analyses

At the item level, less than 6% of all values were missing. As such, missing data was handled *via* pairwise deletion. Preliminary analyses indicated no threats or violations of normality, multicollinearity, or homoscedasticity. PHQ-9 scores were significantly skewed (skewness = 11.08) and kurtotic (kurtosis = 6.72). Additionally, GAD-7 scores were significantly skewed (skewness = 9.15). As a result, Log10 transformations were utilized to more closely approximate a normal distribution. The transformed variables were used in all analyses. However, for ease of interpretation, the non-transformed variable was used in Tables 1, 2, when presenting descriptive statistics and zero-order correlations among study variables. In terms of drop-out, 12% of students reported that they had considered dropping out of medical school in the past month, which the authors found to be significant as categorical data.

**TABLE 1:** Descriptive statistics.

	Mean	Std. deviation	*N*
Gender	0.62	0.485	511
PHQ9_total	6.1255	5.26395	470
GAD7_total	5.3805	4.77567	481
CopingNeg	0.9843	0.92659	511

**TABLE 2:** Means, standard deviations, and zero-order correlations between all variables.

		Gender	PHQ9_total	GAD7_total	CopingNeg
Gender	Pearson correlation	1	0.082	–0.021	–0.017
	Sig. (2-tailed)		0.076	0.640	0.693
	*N*	511	470	481	511
PHQ9_total	Pearson correlation	0.082	1	0.072	–0.025
	Sig. (2-tailed)	0.076		0.132	0.591
	*N*	470	470	442	470
GAD7_total	Pearson correlation	–0.021	0.072	1	0.194**
	Sig. (2-tailed)	0.640	0.132		<0.001
	*N*	481	442	481	481
CopingNeg	Pearson correlation	–0.017	–0.025	0.194**	1
	Sig. (2-tailed)	0.693	0.591	<0.001*	
	*N*	511	470	481	511

**Correlation is significant at the 0.01 level (2-tailed).

Gender coded 0 = Female and 1 = Male; PHQ-9, Patient Health Questionnaire-9; GAD-7, Generalized Anxiety Disorder-7; *p < 0.001.

### Primary analyses

The first hierarchical linear regression was designed to examine the relationship between depression and coping. The first step of the model, which included participant gender, accounted for 0% of the variance in negative coping, *F*(1,468) = 0.14, *p* = 0.705. In the second step of the model, PHQ-9 scores were added accounting for no additional variance in coping, *F* change = 0.53, *p* = 0.466. PHQ-9 scores were not significantly associated with increased negative coping (*B* = –0.03, *t* = –0.73, *p* = 0.466, sr^2^ = 0.00). Therefore, no relationship was found between reported symptoms of depression and the use of negative coping skills among the sample.

The second hierarchical linear regression was designed to examine the relationship between anxiety and coping. The first step of the model, which included participant gender, accounted for 0% of the variance in negative coping, *F*(1,479) = 0.15, *p* = 0.702. In the second step of the model, GAD-7 scores were added accounting for an additional 4% of variance in coping, *F* change = 19.76, *p* < 0.001. GAD-7 scores were significantly associated with increased negative coping (*B* = 0.20, *t* = 4.45, *p* < 0.001, sr^2^ = 0.04). Therefore, a significant relationship was found between individuals reported symptoms of anxiety which were associated with an increase in negative coping skills used. While there were no differences between gender, there was a significant association when examining the sample as a whole.

## Discussion

Results of the current study revealed that students’ depressive symptoms were not associated with an increase in negative coping skills. Although somewhat unexpected, previous findings for depression among Asian Americans have been mixed with some studies showing that sociocultural factors can play important protective roles against negative mood states (Sue et al., 2012; Leong et al., 2013). However, anxiety symptoms were positively associated with an increase in negative coping skills. That is, students who were experiencing anxiety symptoms were more likely to utilize avoidant or negative coping strategies to mitigate the impact of their emotional state. This includes strategies such as engaging in self-harm, over or undereating, smoking cigarettes, using recreational drugs, and consuming alcohol. The utilization of negative coping has been linked to a decrease in academic performance which is concerning given the majority of individuals studied are enrolled in their first and second year of medical school when the curriculum is heavily focused on retention of course materials. In addition, 12% of students who completed the survey had considered dropping out of medical school which is an important prevalence rate for medical school administration to investigate. In order to promote the wellbeing of Asian American medical students during their medical training, it is important to understand how mental health concerns, specifically anxiety, can negatively impact their experience and possibly their professional trajectory. Further, it is important to ensure that Asian American medical students have access to culturally competent providers and culturally sensitive interventions in the academic setting. Interventions to help identify students desire to change, awareness of the problem, and ability to decrease current negative coping skills could be beneficial to the Asian American student population.

In regard to potential limitations of this study, the survey was voluntary and completed on-line by students and may not be representative of the entire population of Asian American medical students currently enrolled. Given the significance of the findings, there may be a greater need in the community then what this particular study captured. The majority of respondents comprised first and second year students which could skew the results. Gaining a balanced sample size across all years of medical school could strengthen the results of this study. The study also assessed student’s intention to consider dropping out of medical school which relates to a potential future behavior. An interesting area of further research would be to assess studies current thoughts about studying less, not attending classes and changes in current behaviors which could affect future performance in medical school. Another potential limitation of the current research was the primary focus on the utilization of negative coping skills rather than assessing if students are using positive coping strategies to mitigate mental health symptoms, which would be helpful to understand in order to build wellness models that align with student’s preferred method for coping with negative emotions. Lastly, students also completed this survey between 2016 and 2017. With the unfortunate increase in discrimination toward Asian American individuals during the last 2 years of the COVID-19 pandemic, the mental health needs of the population surveyed may appear different now with additional societal pressures and social injustice present. Therefore, a survey to examine the current mental health state of this particular group of medical students could be beneficial. In closing, medical schools could strengthen their approach to retain students who are struggling with their mental health, provide supportive interventions when concerns are identified, and demonstrate an understanding and attention to the unique needs of students who identify as Asian American.

## Data availability statement

The raw data supporting the conclusions of this article will be made available by the authors, without undue reservation.

## Ethics statement

The studies involving human participants were reviewed and approved by LSU Health Sciences Center IRB. The patients/participants provided their written informed consent to participate in this study.

## Author contributions

All authors listed have made a substantial, direct, and intellectual contribution to the work, and approved it for publication.
